# The Role of Vaccination Centers in a National Mass Immunization Campaign—Policymaker Insights from the German COVID-19 Pandemic Vaccine Roll-Out

**DOI:** 10.3390/vaccines11101552

**Published:** 2023-09-30

**Authors:** Stella Danek, Dmitrij Achelrod, Ole Wichmann, Falk Schwendicke

**Affiliations:** 1 Charité—Universitätsmedizin Berlin, Corporate Member of Freie Universität Berlin and Humboldt-Universität zu Berlin, Department of Oral Diagnostics, Digital Health and Health Services Research, Assmannshauser Straβe 4-6, 14197 Berlin, Germany; falk.schwendicke@charite.de; 2 Independent Researcher; 3 Immunization Unit, Robert Koch Institute, 13353 Berlin, Germany; wichmanno@rki.de

**Keywords:** COVID-19, mass vaccination centers, health service design, public health service, vaccination strategy, national vaccination campaign, crisis management, pandemic response, lessons learnt, pandemic preparedness

## Abstract

During the COVID-19 vaccination campaign, Germany, like other high-income countries, introduced mass vaccination centers for administering vaccinations. This qualitative study aimed to examine the role that these novel, temporary government healthcare structures played in a mass immunization roll-out and how they can be optimally deployed. In addition, learnings for general emergency preparedness were explored. A total of 27 high-level policymakers responsible for planning and implementing the COVID vaccination campaign at the national and state level in Germany were interviewed in May and June 2022. The semi-structured interviews were analyzed using thematic analysis. Interviewees indicated that mass vaccination structures played an essential role with respect to controllability, throughput, accessibility and openness in line with the key success criteria vaccination coverage, speed and accessibility. In contrast to the regular vaccination structures (private medical practices and occupational health services), public administration has direct authority over mass vaccination centers, allowing for reliable vaccine access prioritization and documentation. The deployment of vaccination centers should be guided by vaccine availability and demand, and vaccine requirements related to logistics, as well as local capacities, i.e., public-health-service strength and the physician density, to ensure effective, timely and equitable access. Improvements to the capacity use, scalability and flexibility of governmental vaccination structures are warranted for future pandemics.

## 1. Introduction

The introduction of the COVID-19 vaccination at the end of 2020 was a ray of hope for ending the COVID-19 pandemic, but also represented a massive organizational and logistical challenge for many public health systems. A vast number of inhabitants needed to be vaccinated both efficiently and at the same time in a controlled manner. A national mass vaccination campaign at this scale had not previously been conducted in Germany.

In Germany, vaccinations are routinely carried out by pediatricians or general practitioners in private (outpatient) medical practices and workplace occupational physicians [1]. However, during the initial phase of the COVID-19 vaccination campaign, only novel mRNA vaccines were available, which were filled in multi-dose vials and demanded ultracool temperatures to remain intact. Furthermore, their availability was initially very limited, making prioritization based on vulnerability and exposure necessary [2]. In order to reach large numbers of people and simultaneously meet these complex logistics requirements, mass vaccination centers were implemented as a new construct in addition to regular vaccination sites [3]. A mass vaccination center is a facility or site not normally used for medical activities (e.g., sport arena or exhibition hall) that has been converted for the large-scale and rapid administration of vaccines [4].

At the start of the national immunization campaign in December 2020, COVID-19 immunizations were carried out exclusively in hospitals (targeting healthcare workers), public mass vaccination centers and their affiliated mobile vaccination teams (targeting mainly nursing homes). The structures routinely providing vaccinations pre-COVID were gradually added from April 2021 onwards [3]. In addition, a legal basis for the participation of dentists, pharmacists and veterinarians in the vaccination campaign was created in December 2021 [5]. Appendix A gives an overview over the campaign’s timeline.

In hindsight, vaccination centers have been referred to as an “essential innovation” for dealing with the pandemic [6]. However, sound scientific research into the role and use of vaccination centers remains limited. Existing studies mostly focus on overviews of different vaccination strategies [4,7,8,9,10], individual operational aspects (e.g., process flows [11], the placement of structures [12,13]) or overall operational lessons learned, mostly drawing on single-site case studies, with a less stringent methodology [14,15,16,17,18,19,20,21,22,23,24]. No retrospective, comprehensive evaluations of the role and use of vaccination centers at the highest level of national strategic coordination have been conducted yet. With regard to Germany, a comprehensive review of the implementation of the COVID-19 vaccination campaign is still lacking.

The aim of this study was to reduce this research gap. The role and use of vaccination centers in mass vaccination campaigns and how they can be optimally used in mass vaccination campaigns, also in comparison to regular structures, were to be examined, drawing on insights from policymakers in the COVID-19 vaccination campaign in Germany. The term policymaker refers to someone “who is involved in establishing policy” [25]. Policymakers were chosen as subjects for their ability to provide privileged, context-specific expert-level knowledge that is hard to access [26,27,28,29]. National- and state-level stakeholders can, furthermore, provide an overarching macro view of the vaccination campaign as a whole and can identify challenges across several vaccination sites, supplementing previous research, which mostly focused on single sites or operational levels in select regions. The results of this study can serve political decision makers and other stakeholders as a basis for the organization of future large-scale vaccination campaigns. It can also provide valuable insights for crisis management in general.

## 2. Materials and Methods

Due to the exploratory nature of the subject, a qualitative approach with semi-structured expert interviews was chosen following the COnsolidated criteria for REporting Qualitative research (COREQ) 32-item checklist for interviews and focus groups from Tong et al. [30] (S1 in Supplementary Materials). The study protocol was reviewed and approved by the ethics committee of Charité Medical University Berlin (application number EA2/051/22). The researchers were guided by a pragmatic interpretive framework [31].

### 2.1. Questionnaire Development

A semi-structured interview guide was developed (S2 in Supplementary Materials), which covered five areas: (i) success criteria for a mass vaccination campaign, (ii) the use of vaccination centers compared to other vaccination structures, (iii) pandemic preparedness and long-term use of vaccination centers, (iv) coordination and organization of the vaccination campaign, and (v) best practices and areas for improvement. The present publication focuses on i, ii, iii, and v. As a basis for the development of the guide, key publications from the World Health Organization (WHO) and the German Federal Ministry of Health (MoH) as well as pertinent academic papers on mass vaccination campaigns and vaccination centers were consulted [3,6,16,32,33]. After review by the study authors and external experts, the interview guide was piloted, with one person familiar with the subject and two people unfamiliar with the subject.

### 2.2. Expert Selection and Data Collection

In Germany, the coordination and implementation of the COVID-19 vaccination campaign was shared between the federal and state levels and select stakeholder groups (more details can be found in the German National Vaccination Strategy [3]). At the federal level, the Federal MoH was in charge of establishing the legal and regulatory framework and overall national coordination of the vaccination roll-out. In addition, the federal government was responsible for vaccine procurement, the allocation to vaccination structures and the physical distribution of the vaccine to the federal states. The Federal MoH received support with logistics from the Federal armed forces, especially in the beginning of the campaign [34,35], and support with the digital capture of vaccination data from the Federal Institute of Public Health, Robert Koch Institute [36]. The federal states were responsible for setting up and operating the public vaccination centers as well as coordinating and implementing the local vaccination campaigns. Representatives of key stakeholder groups, including the National Association of Statutory Health Insurance Physicians (KBV) and the Federal Association for Private Physicians (PBV) for the outpatient medical practices, the Confederation of German Employers’ Associations (BDA) for workplace occupational physicians, the Federal Association of Pharmaceutical Wholesalers (PhaGro) and the Federal Association of German Pharmaceutical Organizations (ABDA) were closely involved in the development and implementation of the vaccination campaign at the federal level.

For our study, we aimed to interview select individuals from all key institutions mentioned above that were responsible for the planning and coordination of the COVID-19 vaccination campaign at the federal or state level: vaccination campaign managers and coordinators from all sixteen German federal states, and managers and coordinators at the federal level, as well as stakeholder representatives at the federal level that could provide expert insights on the role of public vaccination centers in the COVID-19 mass immunization campaign. A combination of purposive and consecutive sampling was used to identify the final interview partners. If the individuals responsible were known, they were contacted directly. Alternatively, liaisons in the respective institutions were asked to name suitable experts.

The experts were divided into two groups upfront: coordinators and stakeholder representatives. Their roles and example expert profiles are described in Table 1. Due to the highly politicized environment, maintaining the anonymity of the interviewees was essential to enable an open exchange. Hence, interviewees were not named by their specific function or name throughout the paper.

The experts were contacted by phone and email between March and May 2022 and invited to participate in the study. Up to four follow-up emails were sent. In two cases, due to rejection and non-response, alternative experts were recruited. Interviews were conducted by SD over four weeks in May and June 2022 in German via video or phone call, recorded, transcribed verbatim electronically using the transcription software Trint in its 2022 Version [37] and de-identified.

### 2.3. Data Analysis

The evaluation was carried out using thematic analysis, according to Braun and Clarke [38,39,40], combining inductive and theory-driven coding. Summary statistics were used to create relevant graphs.

For thematic analysis, the transcripts were read multiple times to identify and collect overarching and recurring categories and themes. Its inductive nature makes thematic analysis “a flexible and useful research tool, which can potentially provide a rich and detailed, yet complex account of data.” [38]. Two authors, SD and DA, independently performed the coding of five expert interviews, filling a coding template and collecting additional salient themes and citations. The coding template focused on the categories relevant to the research questions (theory-driven element); e.g., one category was “national vaccination campaign success criteria”, under which stated themes like “accessibility” or “vaccination rate” were collected with the number of mentions and specific quotes; another category was “deployment criteria”, with themes like “product properties”.

After checking the congruence of the templates and categories, consensus on themes, format and coding framework was reached. To address how the research may be affected by the researchers’ backgrounds [41], reflexivity statements for both SD and DA beyond the COREQ-Checklist can be found in Supplementary Material S1. SD coded the remaining interviews. Select quotes and data, e.g., specific numbers, were returned to participants for review and clarification. Other data points were double-checked using the literature. Once each interview was coded individually (vertical analysis), the findings were compared across interviews (horizontal analysis) [42]. The results were summarized, discussed iteratively among all authors and refined until consensus.

## 3. Results

A total of 27 of the 29 contacted experts participated in the interviews (Table 1).

Twenty-two participants were coordinators at a federal or state level who coordinated either the overall COVID-19 crisis response within the healthcare realm, the overall COVID-19 vaccination campaign or various aspects of the vaccination roll-out, e.g., the national-vaccination-campaign coordinator in the federal MoH, the national vaccine distribution center manager, the division head of a state MoH responsible for COVID-19 crisis coordination. All sixteen federal states of Germany were represented.

Five interview participants were key stakeholder representatives involved in the implementation of the vaccination campaign at the federal level, e.g., representatives of the national association of statutory (public) health insurance physicians and the private physicians responsible for coordinating the integration of outpatient medical practices in the vaccine roll-out or the head of the federal association for pharmaceutical wholesalers supporting vaccine distribution.

The interviews lasted on average 58 min (min. 30–max. 97). For more details, see Supplementary Material S1.

**Table 1:** Overview of interview partners and assignment to expert groups.

Description	Example Expert	Number of Interviews			
		Federal Level	State Level	Total	Planned
**Coordinator** (management, planning, implementation)		5	17	22	24
Coordinators defined the general requirements (legislation) for the implementation of the vaccination campaign and/or planned and coordinated the operational implementation of the vaccination campaign in their respective realm	National vaccination campaign coordinator at federal level in the Federal Ministry of Health Managing director of the national vaccine distribution center for COVID-19 vaccines Head of the vaccine administration data collection project at federal level in the Robert Koch Institute Head of a federal state’s COVID crisis management team in a state Ministry of Health Manager of COVID vaccination centers of a federal state				
**Key stakeholder representative**		5	/	5	5
Representatives of the stakeholders involved in the implementation of the vaccination campaign contributed to the general vaccination campaign roll-out and planning, operation of vaccination centers and coordinated the operation of other vaccination structures.	Representative for outpatient public medical practices of the National Association of Statutory Health Insurance Physicians (KBV) Representative for outpatient private medical practices from the Federal Association of Private Physicians (PBV) Representative for pharmacists from the Federal Association of German Pharmaceutical Organizations (ABDA) Representative of pharmaceutical wholesalers from the Federal Association of Pharmaceutical Wholesalers (PhaGro) Representative for workplace occupational physicians from the Confederation of German Employers’ Associations (BDA)				

Overall, the themes could be clustered along four areas:

1. Target criteria for a successful national pandemic vaccination campaign;
2. Role and optimal deployment of state vaccination centers;
3. Additional take-aways for best practice of vaccination center roll-out;
4. Post-pandemic: Operational readiness and transfer of the vaccination-center concept.

### 3.1. Target Criteria for a Successful National Pandemic Vaccination Campaign

**Figure 1:**
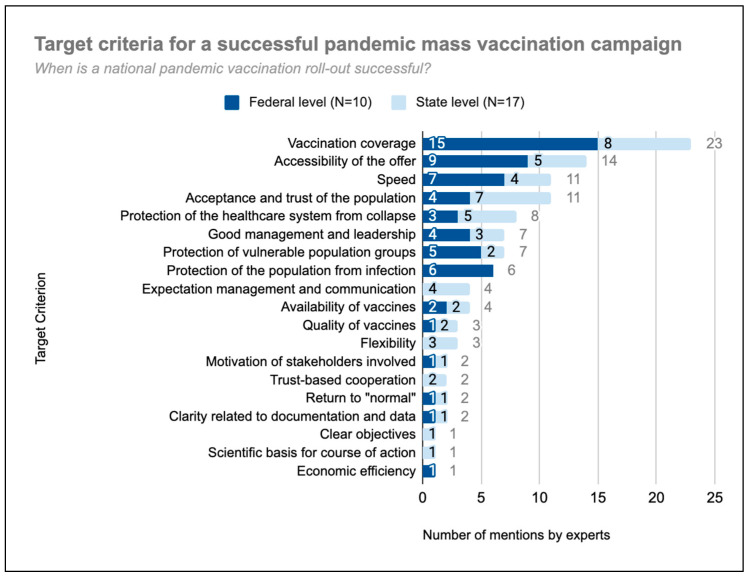
Target criteria for a successful national mass vaccination campaign based on frequency of mention by experts (N = 27).

Experts named a total of 19 criteria that make a national pandemic vaccination campaign successful (Figure 1). Every expert named 4 criteria on average (min. 1–max. 10). Vaccination coverage was deemed the most important success factor by far (23 mentions).

The accessibility of vaccination offers, i.e., the ability of every inhabitant to obtain a vaccination, minimizing barriers to access, was also named by more than half of the experts. More than a third named speed (“*as many people as possible in a short time*.”) (federal-level stakeholder representative) and acceptance and trust of the population. “*Of course, we want people to get vaccinated. But having an educated population that deliberately decides not to get vaccinated could also mean that the campaign was successful. The success consists in the population being knowledgeable and informed about the vaccines*”. (federal-level coordinator).

Only one expert named cost-effectiveness as a success criterion. Two federal-level coordinators even refuted the relevance of economic efficiency within a pandemic: “*I think economic factors play a minor role in the vaccination campaign.*” “*Economic efficiency was not always a top priority in the context of the COVID pandemic*”.

For additional quotes, view Supplementary Material S3.

### 3.2. Role and Optimal Deployment of State Vaccination Centers

All 27 experts agreed that the vaccination centers made an important contribution to the COVID-19 vaccination campaign in Germany and to the achievement of the aforementioned goals. Experts called government vaccination centers “*without alternative*” (state-level coordinator), “*essential*” (state-level coordinator, federal-level coordinator), and “*significant*” (federal-level stakeholder representative). Experts pointed out that medical-practice capacities were not sufficient to quickly vaccinate the entire population (federal-level coordinator), while concurrently vaccination centers alone could not have facilitated the vaccine roll-out (federal-level stakeholder representative). Two state-level coordinators and two stakeholder representatives initially opposed the use of vaccination centers, but in retrospect rated it positively.

#### 3.2.1. Advantages and Disadvantages of Public Mass Vaccination Centers

**Figure 2:**
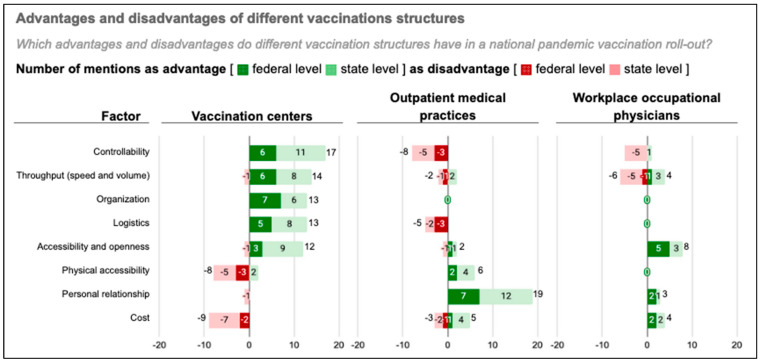
Advantages and disadvantages of state mass vaccination centers compared to medical practices and workplace occupational physicians according to the frequency of mention by experts (N = 27).

The most frequently mentioned advantages and disadvantages of different vaccination structures focused on eight factors (Figure 2). Controllability (N = 17), high throughput (volume and speed of vaccination) (N = 14), efficient logistics (N = 13), openness/accessibility (N = 12) and organization (N = 13) were highlighted as advantages of the vaccination centers. Three of these (throughput, low-threshold access, and organization) were also mentioned as top 10 objectives of the vaccination campaign. Disadvantages noted were cost (N = 9) and physical accessibility (N = 8).

All 27 experts commented on vaccination centers, 25 on medical practices, and 22 on workplace occupational physicians (Figure 2). Ten commented on mobile vaccination teams and few experts commented on the advantages and disadvantages of the vaccination structures added towards the end of the pandemic vaccination campaign—i.e., pharmacies, dentists, and veterinarians. One expert commented: “*People who are not yet vaccinated [more than a year after the start of the vaccination campaign] will not be convinced by talking to a dentist, veterinarian or pharmacist.*” (federal-level stakeholder representative).

##### Controllability

Thirteen experts referred to a lack of controllability as a key disadvantage of medical practices and workplace occupational physicians. This was linked, among others, to a lack of transparency about their actual participation and capacities (state-level coordinator) and a lack of direct government authority on their use (state-level coordinator). It contrasts the positively noted ability to actively manage the vaccination centers. On this theme, several interview partners emphasized the reliability of vaccination centers related to implementing the mandated prioritization policy (N = 16) and adequately documenting and reporting vaccinations at the national level (N = 5). Failure to comply with prioritization has been cited as a downside of involving medical practices in the roll-out and a potential threat to equitable vaccine access. “*Without a state-organized structure, there would have been more problems with fair access*” (state-level coordinator). “*The prioritization would have failed in the medical practices”* (federal-level stakeholder representative).

##### Throughput (Speed and Volume)

Some experts attributed the high throughput of vaccination centers to focused and standardized processes: “*The vaccination centers did nothing else [apart from vaccinating] all day. This also allows you to work efficiently*.” (state-level coordinator). However, in medical practices, familiarity with patients allowing shorter briefings could save time (state-level coordinator). There was no agreement on throughput for workplace occupational physicians. The role of workplace physicians was generally commented on sparsely, but a recurrent theme was the gap between their theoretical potential and their actual contribution to the COVID-19 vaccine roll-out. This is also reflected by four experts rating their throughput positively and four negatively. Workplace occupational physicians can theoretically vaccinate large cohorts, but in retrospect, according to nine experts (eight of them coordinators at state level), it had “*little relevance*” and “*fell short of expectations”*. Reasons given included large differences in performance between providers, and success at “*big committed companies*”, but only few big companies involved, “*often doctors in secondary employment*”, a late involvement in the campaign, poor vaccine supply, and a lack of compensation.

One federal-level stakeholder representative commented overall: “The speed of different structures is difficult to assess, [… it is] distorted by distribution and vaccine availability at different points in time”.

##### Logistics

Delivery to a limited number of circa 430 vaccination centers is “*more stable*” (federal-level coordinator) than delivery to an estimated 60,000 scattered private medical practices. Centers can pool demand, e.g., through registration systems (state-level coordinator), multi-dose vials can be used up more efficiently, reserves can be centrally mandated to absorb delivery irregularities (state-level coordinator) and complex vaccines are more adequately handled and stored (state-level coordinator). In particular, the potential waste of vaccines was identified as a challenge in medical practices (federal-level coordinator).

##### Accessibility and Openness

At government vaccination centers, every inhabitant is guaranteed to receive a vaccine without necessarily needing a family physician (young, migration background, living in areas of low physician density, marginalized groups). Barriers to access are lowered through spontaneous walk-in vaccination initiatives, e.g., designated “open house” days (state-level coordinator); the fast allocation of appointments (federal level stakeholder representative); translation services (state-level coordinator); targeted offers, e.g., clown-accompanied child vaccination days; and continuous, everyday offers during off-peak times (after work).

In contrast, medical practices cannot continuously vaccinate, since regular medical care must be maintained and resources are already strained (federal-level stakeholder representative, state-level coordinator, federal-level coordinator). Yet, two experts mention the possibility of combining the COVID vaccination with regular physician visits as low-threshold elements (federal-level coordination; state-level coordinator). Eight experts also rated workplace vaccinations as low-threshold interventions, as they can be integrated into work routines.

##### Physical Accessibility

All state-level experts, who lamented proximity issues for vaccination centers came from non-city territorial states with large rural areas (note: in Germany, three of the sixteen federal states are city states: Berlin, Hamburg and Bremen). In this context, ten experts emphasized specific advantages of state mobile vaccination teams for “*reaching people where they are*” (federal-level coordinator) in their habitual environment, especially for vulnerable or disadvantaged population groups (long-term care facilities, language barriers, religious communities, immobile individuals at their home). Where mentioned (N = 6), experts seemed to agree that medical practices, as a distributed structure, are easier to reach. “*The distance between where you live and the vaccination site is of course much better in the case of medical practices*”. (state-level coordinator).

##### Personal Relationship

Nineteen experts named themes surrounding personal relationships, trust, and an individualized approach to the patient as an advantage of medical practices. The familiarity of patients facilitates prioritization, individual consultation, and a more targeted explanation of possible side effects (five experts at the federal and state level). The personal approach and trust-based relationships could also help reach vaccine skeptics (two state-level coordinators). Simultaneously, the lack of personal ties did not necessarily appear to be unfavorable for vaccination centers. Only one state-level coordinator suggested so. In contrast, one federal-level stakeholder representative saw anonymity as an advantage. “*The threshold for getting vaccinated is lower because you don’t have to reveal everything like you would with a family doctor*”. Another state-level coordinator noted that “*there was no feedback to suggest that there was less trust in the vaccination centers*”.

##### Cost

While cost-effectiveness was only mentioned once as a success criterion (see Section 3.1), nine experts named high cost and effort as inconveniences of vaccination centers. The vaccination centers are a “*really expensive structure*” (state-level coordinator). The assembly and dismantling are labor-intensive and the structure is “*rigid*” (state-level coordinator), “*large and sluggish*” (federal-level coordinator). Experts noted that for medical practices and workplace occupational physicians, costs are lower as the infrastructure is already established. With respect to occupational health physicians, companies themselves bore the vaccination costs (federal level stakeholder representative, state-level coordinator). One federal coordinator commented on the financial aspects overall: “*Yes, vaccination centers are, of course, incredibly expensive, but that’s always the issue with preventive measures. They’re expensive, but they also protect. A society has to be willing to spend money on something like that.”* Along those lines, a state-level coordinator noted: “*The vaccination centers are a public service. They have resources in standby that cannot be economically viable, because they are supposed to cover a delta [of vaccinations] in order to provide a [broad] offer*.” A state-level coordinator noted that the high remuneration for physicians in vaccination centers (around EUR 80–175 per hour [43,44]) shifted market dynamics, as it was more profitable for physicians to vaccinate in centers than offer COVID vaccinations in their own practices. At the same time, the remuneration per COVID 19-vaccination administered within the practices was also considerably high at EUR 28–36 compared to EUR 8 for administering a flu shot [45,46].

##### Symbolism

Four experts additionally discussed the themes visibility and symbolism. Vaccination centers can serve as fixed, visible access points, associated with vaccinations (two state-level coordinators). “*[The vaccination centers] were a symbol visible to the outside world: Something important is happening here! […] We have to tell a story.*” (state-level coordinator). The placement in central locations is a good way to reach additional people. Getting vaccinated by workplace occupational physicians could also have a “*snowball effect*”: “*If my buddy on the assembly line goes to get vaccinated, I’ll just go with him*” (federal-level stakeholder representative). Workplace occupational physicians play a crucial role in maintaining industry and critical infrastructure (federal-level stakeholder representative).

#### 3.2.2. Deployment of Public Vaccination Centers for National Pandemic Vaccination Roll-Out

##### Deployment Criteria

The experts named topics related to pandemic conditions (N = 23), product properties (N = 15) and local capacities (N = 16) as the main criteria for establishing vaccination centers and other government vaccination structures (Table 2).

The experts named topics related to pandemic conditions (N = 23), product properties (N = 15) and local capacities (N = 16) as the main criteria for establishing vaccination centers and other government vaccination structures.

According to the interviewees, the prioritization mandated by vaccine shortage (demand higher than supply) is the key criterion for using government vaccination structures. Pandemic vaccines are a scarce resource that needs to be closely managed. Referring to distribution challenges with H1N1-vaccines in 2009 [47], one expert noted: “*the established system [i.e., decentralized private medical practices] does not make sense in such a situation. We already had that experience in 2009.*” (state-level coordinator). Two state-level coordinators noted that before involving the physician offices, there would need to be enough vaccines to distribute to all offices wanting to participate in the roll-out.

Complex product properties relate to container size, storage and transport conditions. A federal-level stakeholder representative noted that as long as vaccines are only available in multi-dose vials, a full transition to outpatient practices is difficult due to potential waste.

Local circumstances and capacities included the following criteria: local infrastructure in relation to rural versus urban areas and the corresponding access routes, the burden on regular outpatient care structures, the local physician density and the strength of the local public health service. Several participants mentioned the already-overstretched resources of medical practices (N = 4). Others commented that vaccination centers can offer relief for other structures (N = 4) and fill gaps in areas with low physician density (N = 3).

In addition, state-level coordinators mentioned the importance of the 50% financial subsidy [3] from the federal government (N = 4) and political and social pressure (N = 3) for establishing or dismantling mass vaccination centers. The discontinuation of federal funding support was noted as a reason for dismantling state vaccination centers.

##### Transition Timing during the COVID-19 Vaccination Roll-Out

In April 2021, i.e., five months after the start of the national immunization campaign with government mass vaccination centers and mobile teams, medical practices were involved in the COVID-19 vaccination roll-out in Germany (Appendix A). There is no consensus among experts as to whether this was the right time to transition from central to more decentralized provision. Six commented that it was the right time, four too early, and four too late. The votes were mixed from all expert types.

##### The Changing Role of Government Immunization Services over the Course of a Pandemic

The discussions also showed that the role of vaccination centers and government immunization services shifted from an exclusive, central role to a supporting role over the course of a pandemic vaccination roll-out. Eight experts specifically noted that vaccination centers were most relevant in the beginning of the pandemic vaccination campaign. As prioritization is progressively loosened, vaccines become more manageable in outpatient settings and demand lowers, vaccination centers become a “supporting pillar” (state-level coordinator). Factors surrounding local capacities become more relevant for operating additional public immunization structures. Yet, even if demand decreases, the state is liable for reaching all population groups (see accessibility in Section 3.2.1). *“The ultimate goal is to make vaccinations available as low-threshold and broadly as possible.”* (state-level coordinator). Concurrently, a federal-level coordinator noted a limit to additional government offers: *“At some point, the whole thing reaches a limit. You don’t have to follow people around with a syringe 15 times. If they still say no, then at some point you need to accept that part of the population likely will not get vaccinated”*.

### 3.3. Additional Take-Aways for Best Practice Vaccination-Center Roll-Out

Beyond the aforementioned factors, some recurring themes provided insights on optimal vaccination-center roll-out:

- The flexibilization of government vaccination structures;
- The better use of available vaccination capacities;
- The broad involvement of stakeholders and expertise.

#### 3.3.1. Flexible Design of Government Vaccination Structures Adapted to Circumstances

Overall, experts agreed that the government vaccination offer could be diversified and adapted more specifically to different circumstances. Seven experts specifically named simplification, flexibilization and less “*German perfectionism*” as areas of improvement. The vaccination centers should be “*smaller, more mobile, more punchy*” (federal-level coordinator). While mass vaccination centers appear suitable for both rural and urban areas, they require mobility (federal-level stakeholder representatives). A state-level coordinator noted that in sparsely populated areas, building large sites is not worthwhile, because they need “*a certain catchment area*.” (state-level coordinator). Another state-level coordinator noted that when demand drops, a switch from “come-here” to “go-to” structures is necessary, i.e., from rigid, locally fixed centers to mobile, outreach units targeting living quarters (e.g., long-term care facilities, social hotspots) and everyday life (e.g., shops, gastronomy, sport clubs). The structures should complement each other (state-level coordinator). Hence, different designs beyond mass vaccination centers and mobile vaccination teams are necessary, e.g., smaller, temporary pop-up stations, as seen towards the end of the pandemic vaccination campaign. Another example given was a barrack close to Berlin where festival tents were used as vaccination booths rather than building complex structures with removable walls. After the official closing of vaccination centers in September 2021, several experts mentioned diverse configurations of government vaccination sites.

#### 3.3.2. Scalability and Optimized Use of Available Vaccination Capacities

Several experts (N = 11) also noted that the capacity of the vaccination centers was not fully taken advantage of, and that scalability of government vaccination sites could be improved. Better data, digital systems, centralized appointment and capacity management would facilitate needs-based planning. As one federal-level stakeholder representative notes: “*You have to take advantage of all the opportunities offered by digitalization*”. Additionally, scalability could be improved through more flexible staffing and rental contracts.

#### 3.3.3. Broad Stakeholder Involvement and Use of Domain Specific Expertise

Furthermore, the broad, multidisciplinary cooperation at eye level between various actors including aid organizations, different sections (from education to economy) and levels (from federal to local) of the public administration and the private sector, was praised. Specifically, the use of “external expertise” was mentioned as the best practice (N = 6). This included, for example, working with trade fair constructors and the event industry to quickly build and manage vaccination centers as well as the close exchange with local pharmacist chambers or building an alliance across aid organizations, as was the case in Berlin.

### 3.4. Post-Pandemic: Operational Readiness and Transfer of the Vaccination Center Concept

Experts agreed that in the long run, vaccinations against COVID-19 would take place in the regular (privatized) outpatient structures and that after the pandemic, the vaccination center structure, with its “very specific purpose” (state-level coordinator), should be abandoned. It was also noted that once vaccination centers are dismantled, rebuilding is challenging, particularly due to the reactivation of leases and staff contracts (state-level coordinator).

Nine experts noted that the vaccination center infrastructure could be used in the medium term for initial medical examinations and for the accommodation of Ukraine war refugees who came to Europe at the time of the survey. Most of the experts (N = 16) believed that in the long run, the vaccination-center experience would mostly serve in a figurative sense, as a helpful learning experience for future crisis events characterized by time pressure and mass incidence, e.g., climate catastrophes, flooding, poliomyelitis outbreak, bioterror, nuclear attack. Additionally, five experts emphasized that states had learned to set up massive operations on extremely short notice.

There was no agreement among the experts on the optimal procedure for maintaining vaccination structures or on measures that ensure operational readiness in the event of a future disaster. The creation and updating of blueprints, checklists and crisis plans, emergency training, materials storage and identification of suitable locations were discussed. Four experts mentioned staffing as a particularly challenging factor. An overview of potential measures mentioned can be found in Table 3.

Several experts emphasized the significance of state structures and the need to strengthen the public health service in Germany as the third pillar of the health system post-pandemic after decades of cutback. The state has to “care for its sheep” (federal-level coordinator) and the pandemic served as an eye opener on this end. “*The pandemic has clearly shown that a strong public health service is essential. We can’t just dump everything on the medical practices, because it’s in our all’s interest […] that we can really reach all population groups*”. (state-level coordinator).

Overall, experts stressed the importance of conducting Lessons Learned processes. At the time of the interviews, only one state-level coordinator stated that an evaluation had been carried out; three others stated that an evaluation was currently being conducted.

## 4. Discussion

### 4.1. Principal Results

The study draws on insights from key high-level public health experts, policymakers and stakeholder representatives at the national level and from all sixteen federal states that were intimately involved in the roll-out of the COVID-19 vaccination campaign in Germany. It finds that publicly administered vaccination centers can play a crucial role in pandemic mass immunization campaigns characterized by the need for speed, vaccine availability constraints and an accessibility mantra. The advantages of vaccination centers appear to be in line with the main success criteria of a mass vaccination campaign.

While several experts (N = 14) noted throughput, in terms of high volumes and/or speed, as a key advantage of vaccination centers, the available literature on the topic is inconclusive. An analysis from the beginning of the roll-out [48] suggests that the speed of the German vaccination campaign increased when medical practices joined, but this may be linked to increased time between doses rather than differences between vaccination centers and medical practices. From a user perspective, a study of all Berlin-based vaccination centers showed that long wait times and overall duration were emphasized as negative aspects of vaccination-center interaction [49], while a survey comparing 10 GP offices to two vaccination centers in Saxony suggests that wait times were longer in GP practices [50]. Yet, vaccination centers are a “one-stop shop” or “single purpose structure” focusing on just one task, which should provide an opportunity to optimize the process and allot for faster vaccination than in a generalist setting. There is also debate around the economics of vaccination centers, as already emerged from the expert interviews. No conclusive quantitative assessment could be found in the literature. Friendliness, which the Berlin study found was an outstanding feature for mass vaccination centers from a user perspective [49], was not prominently debated by experts.

Another crucial question concerns the right point in time for the transition from central to more decentralized vaccine roll-out, including regular vaccination structures. The study found pandemic conditions surrounding vaccine scarcity and excess demand as well as logistical and product criteria to be key. This was in line with other studies and policy papers [3,6,16,32,33]. However, the study revealed new insights concerning the importance of capacity-related criteria as additional consideration for deployment, as well as the changing role of government vaccination centers, in order to ensure speed and accessibility throughout the pandemic vaccination campaign even after decentralization. Grimm, Lembcke and Schwarz [51] also include vaccine capacities in their trifactor criteria of vaccination campaign progress (vaccine capacities, availability and uptake). Overall, there was no agreement on transition timing. It appears that countries like the US, Israel and the UK succeeded in abandoning the prioritization and including medical practices earlier on [48].

For future pandemics, improvements in scalability and better capacity management, as seen towards the end of the roll-out, are warranted. Efficient resource use is crucial during a pandemic, where resources, financial and human, are already strained. However, for better, real-time capacity planning and forecasting, reliable data and modern digital tools are necessary. A review from the Region Westfalen-Lippe suggests the unpredictability of vaccination delivery schedules was a challenge for the optimal use of vaccination capacities [21]. Despite some successful initiatives, digitalization, especially of public services, remains a key challenge in Germany, but is the cornerstone of proper monitoring and forecasting. It could also help improve expectation management and communication, which are known success criteria of managing the pandemic. Additionally, the theme of public infrastructure throughout the studies serves as a reminder of the importance of government crisis response structures to ensure a swift, controlled response as well as fairly distributing public goods. This nicely reflects the current public debate around reinforcing defense capabilities, strengthening public health services and the national reserve for healthcare protection. Secondly, the cooperation of different stakeholders and the use of domain-specific expertise is the key for a successful mass immunization campaign. The cooperation of different stakeholders, here, medical practices, clinics and the public health services, was also cited as key for success in a case study on the COVID-19 vaccination campaign in Baden-Württemberg [52].

### 4.2. Strengths and Limitations

This study had some limitations. Firstly, qualitative thematic analysis limits the possibility of applying formal statistical methods and encoding is inherently interpretive, limited by the biases of the evaluators and interview participants as to what and how they want to disclose results. To improve reproducibility and reliability, we drew on multiple forms of validation including researcher triangulation, the verification of details with participants and the gray literature review. We further conducted this research with a multidisciplinary team composed of experienced researchers with diverse backgrounds including health policy, health economics, medicine, epidemiology, data science and health services research. Two members of the research team are deep experts on the German vaccination campaign, whereas two are not. Through multiple iterations, this allowed for reflection on possible experiential and discipline-related thought patterns. Finally, the versatility and flexibility of thematic analysis captures human interaction and is particularly suitable for exploratory studies such as the present one in order to identify, analyze and report patterns and themes [38,53].

Secondly, while interviewees were intimately involved in the organization or implementation of the COVID-19 vaccination campaign, not all were at the exact same level of seniority within their respective institutions. Additionally, observations within the German capital may be overrepresented, since two state-level coordinators and all federal-level experts except for one work in Berlin. At the same time, policymakers from all sixteen federal states participated, giving an overarching view of a national campaign and supplementing previous research, which mostly focused on single sites or operational levels in select regions.

Thirdly, recall and desirability bias are a common concern in interview-based research. Furthermore, some experts represented specific interest groups and all were, by their nature as coordinators and stakeholders, intimately involved in the success or failure of the campaign. Another concern noted regarding interviews conducted during the pandemic is the swiftly shifting context [54,55]. To reduce recall bias and issues stemming from timeliness, interviews were conducted within a short four-week time frame during a relatively calm point in the pandemic, after elections, where the administrators in charge during the pandemic were still in office and could recall lessons with little hindsight. To reduce desirability bias and allow for open, even critical, discussions on politically charged topics, interviews were anonymized. Furthermore, ten experts were personally known to the interviewer. In order to reduce interviewer bias and improve comparativeness, the same interview guide was used across participants. Despite using an interview guide, questions and contexts of interviews can vary [56].

Finally, the findings arose from a specific healthcare system context: Germany has a strong primary healthcare system where vaccinations typically take place in private, decentralized outpatient medical practices. Furthermore, Germany does not have a central vaccination registry. Nonetheless, some findings may be applicable for countries and contexts with stronger state immunization programs.

### 4.3. Future Research Needs

To our knowledge, this is the first study to provide a broad, national-level assessment on the role of vaccination centers in a national mass vaccination campaign. It is also the first national-level lessons-learnt study on the COVID-19 pandemic in Germany, drawing on insights from high-level policymakers and other stakeholders involved in vaccination roll-out and crisis management. The study adds a high-income-country perspective to an existing body of the literature on lessons learnt from mass vaccination campaigns at a national level, which previously focused mostly on low- and middle-income countries [57,58,59]. International comparisons of the role of and deployment criteria for mass vaccination centers, as well as further studies drawing on vaccine recipient and operator perspectives across vaccination centers, are needed. Future studies should also provide a quantitative assessment regarding operational efficiency, resource use and the cost of different vaccination structures.

## 5. Conclusions

Overall, the experts interviewed believe that vaccination centers played a crucial role in the national COVID-19 vaccination roll-out in Germany and can be especially advantageous with regard to control in a situation handling a scarce and complex vaccine, as well as with regard to accessibility to ensure equitable access for all inhabitants in times of crisis. It appears that for future crisis-response scenarios, government vaccination structures need to be more adaptable to the specific context. Vaccination centers also highlight the importance of governmental crisis-response structures for a managed response, but can also serve as a symbol of government action in central locations, a “cue to action” [60] for citizens to take up vaccination and a sign of “hope” [61] during a traumatic mass event.

## Figures and Tables

**Table 2:** Criteria for establishing additional centrally managed, state vaccination structures.

Factors Increasing the Need to Establish Centrally Managed Government Vaccination Structures beyond Regular Primary Care Infrastructure	
**Pandemic conditions** (N = 23) *	Limited **availability of vaccines** paired with high **demand** (fair distribution challenges, need for prioritization of select population groups, lack of ability to provide all outpatient practices with vaccines) Need for tighter **control and follow-up** (limited knowledge about side-effect profiles of novel vaccines, accuracy of vaccination coverage data, detection of vaccination gaps, evolving virulence, and infection rates, need for security, quality and standardization, cushioning of delivery irregularities) **Political and public pressure** (symbolism of activity, need to comply with public and political will, ability to provide information)
**Product properties and logistics** (N = 15) *	Complex **storage and transport conditions** (Galenics/vaccine stability, cold-chain maintenance, complex preparation) **Vial contents** (multi-dose vials instead of pre-filled syringes)
**Local circumstances and capacities** (N = 16) *	Overstretched **resources** of regular outpatient healthcare structures Low **local physician density** Weak **public health service** Rural vs. urban **infrastructure**/large **distance** to closest vaccination site

* Number of experts mentioning this item (total of N = 27).

**Table 3:** Collection of possible preparedness measures for the rapid establishment of crisis response structures mentioned by experts (not exhaustive).

Operational Readiness Measures for the Rapid Establishment of Crisis Response Structures	
**Processes**	**Evaluate processes** employed during the COVID-19 pandemic **Create and update** blueprints, checklists and **contingency plans** **Outline sample process chains and structures**, e.g., clear responsibilities, especially with regard to the demarcation of state governments vs. the federal government, communication chain, registration and documentation **Create templates** for public tenders and sample models
**Locations/properties**	**Designate adequate locations** and prioritize the order of use Plan with **more flexible structures** (large structures such as exhibition halls are not absolutely necessary) **Reserve space within the public health service** (small vaccination centers) **Cooperate** with the city and municipality council for the selection of properties
**Staff**	**Create profile archetypes** with typical qualifications and responsibilities **Train management staff** (also pre-crisis) **Train professional preparedness teams** **Identify potential staffing pools**, e.g., list of retired doctors, event and catering industry, trade shows, hospitality **Strengthen health skills** of emergency/crisis response teams as well as the general public **Conduct emergency drills**
**Materials/** **technology**	**Establish a state material warehouse** for storage of Universally usable material, e.g., partitions, chairs, tables, sirens, … Medical materials, e.g., personal protective equipment, vaccination kits (syringes, needles, …), vaccines (e.g., smallpox vaccine against a background of monkeypox) Storage of technical equipment (i.e., computers, tablets, …) not recommended due to rapidly changing technological standards

**Figure A1:**
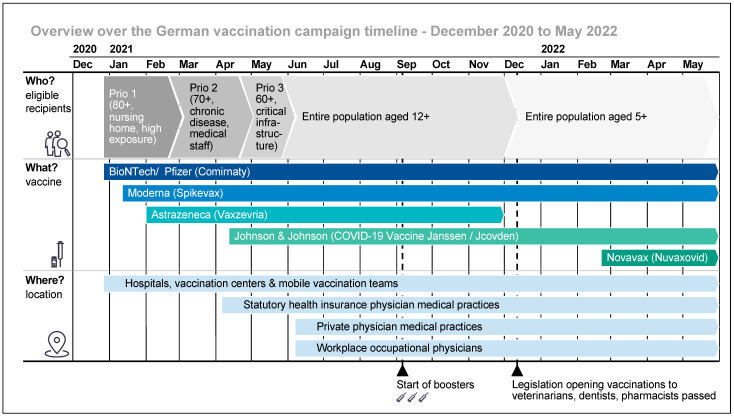
The national COVID-19 vaccination campaign in Germany over time from December 2020 to May 2022.

## Data Availability

The raw data are unavailable due to privacy restrictions.

## Supplementary Materials

The following supporting information can be downloaded at: https://www.mdpi.com/article/10.3390/vaccines11101552/s1, S1: COREQ-Checklist and reflexivity statements; S2: semi-structured interview guide for crisis/vaccination roll-out coordinators at federal- and state-level ministries of health and stakeholder representatives at federal level in Germany; S3: additional quotes from federal and state-level coordinators and federal-level stakeholder representatives on the role of vaccination centers in the German COVID-19 vaccination roll-out.

## Appendix A

SD created this overview, consulting various publicly available sources including national-level policy papers, legislative texts and news coverage [3,62,63,64,65,66,67,68,69,70]. Additionally, SD consulted with experts at the Federal Ministry of Health (vaccination-campaign coordination unit, vaccine-logistics coordination unit, and infectious diseases division), the German Armed Forces (logistics coordination), and the Robert Koch Institute (data management coordination and immunization unit) to close data gaps and review the overall timeline.
