# Supplementary material for: The Role of Vaccination Centers in a National Mass Immunization Campaign—Policymaker Insights from the German COVID-19 Pandemic Vaccine Roll-Out
Source: Vaccines (Basel). 2023 Sep 30;11(10):1552. doi: 10.3390/vaccines11101552 (PMC10611148; doi:10.3390/vaccines11101552)
Supplement: Supplementary file 1 [file vaccines-11-01552-s001.zip › S1_Supplement_S1_vaccines-2600323_edited - revisions 1_vF_proof.pdf]

S1. COREQ-Checklist and reflexivity statements

COREQ-Checklist

Developed from: Tong A, Sainsbury P, Craig J. Consolidated criteria for reporting qualitative research (COREQ): a 32-item checklist for interviews and focus groups. International Journal for Quality in Health Care. 2007. Volume 19, Number 6: pp. 349 – 357

No. Item	***Guide/Recommendation***	***Description***
Domain 1: Research team and reflexivity		
Personal Characteristics		
1. Interviewer/ facilitator	*Which author/s conducted the interview or focus group?*	SD
2. Credentials	*What were the researcher's credentials? E.g. PhD, MD*	Undergraduate studies in Political Science, History and Economics; graduate studies in Health Policy and Health Economics; professionally active in health policy and healthcare innovation for 7+ years
3. Occupation	*What was their occupation at the time of the study?*	Doctoral researcher
4. Gender	*Was the researcher male or female?*	Female
5. Experience and training	*What experience or training did the researcher have?*	7+ years working in health policy and healthcare innovation, 2 of them as research assistant and policy advisor conducting health policy and health economics research.<br>Experience working in national public administration on vaccine campaign coordination and organization; experience studying vaccination centers and national vaccination campaign implementation.
Relationship with participants		
6. Relationship established	*Was a relationship established prior to study commencement?*	Some interview partners were known beforehand, through a prior professional working relationship (N= 10 of 27)
7. Participant knowledge of the interviewer	*What did the participants know about the researcher? e.g. personal goals, reasons for doing the research*	Reasons for doing the research: scientific study, gaining an understanding of the optimal role and use of vaccination centers, contributing to the pandemic lessons learnt process
8. Interviewer characteristics	*What characteristics were reported about the interviewer/facilitator? e.g. Bias, assumptions, reasons and interests in the research topic*	Reasons and interests in the research topic: scientific study, gaining an understanding of the optimal role and use of vaccination centers, contributing to lessons learnt from the pandemic
**Domain 2: study design**		
Theoretical framework		

9. Methodological orientation and Theory	What methodological orientation was stated to underpin the study? e.g. grounded theory, discourse analysis, ethnography, phenomenology, content analysis	Thematic Analysis according to Braun and Clarke
Participant selection		
10. Sampling	How were participants selected? e.g. purposive, convenience, consecutive, snowball	A combination of purposive, snowball and consecutive sampling was used to identify the final interview partners.<br>Purposive: Known focal points for the pandemic response from the health ministries of the 16 federal states, the federal level and representatives from other involved organizations were directly contacted.<br>Snowball: where the responsible coordinators for the COVID-19 vaccination campaign coordination were unknown, known focal points were asked to provide adequate contact persons.
11. Method of approach	How were participants approached? e.g. face-to-face, telephone, mail, email	E-mail and telephone.<br>29 selected experts were contacted for participation. A follow-up was necessary for 18 experts. Experts were contacted by email up to 4 times.
12. Sample size	How many participants were in the study?	27 participants
13. Non-participation	How many people refused to participate or dropped out? Reasons?	1 person refused to participate (citing concerns regarding anonymity), an alternative participant from the jurisdiction was recruited.<br>1 person first accepted to participate, but then stopped answering.<br>Otherwise, when a contacted person or entity did not have the required expertise, the contacted entities referred the researcher to an adequate expert (refer to participant selection above)
Setting		
14. Setting of data collection	Where was the data collected? e.g. home, clinic, workplace	27 by videocall (25) or phonecall (2). All took place at the work place.
15. Presence of non-participants	Was anyone else present besides the participants and researchers?	No. 26 calls were exclusively bi-lateral (researcher and expert). In 1 circumstance a silent observer (student) was present in an expert's office.
16. Description of sample	What are the important characteristics of the sample? e.g. demographic data, date	Interviews took place between 04 May 2022 and 01 June 2022.<br>All 16 federal states were still offering government vaccination services, proper mass vaccination centers were still in active use in 4 federal states.<br>The COVID-19 pandemic vaccination campaign had started in December 2020. At the time of data collection daily vaccination rates were comparatively low with an expected potential surge in rates in the fall.

		10 interview participants were female, 17 male.<br>Participants were in varying positions of seniority in public administration or professional stakeholder associations ranging from policy officers leading the vaccination campaign coordination in executive units to managing directors of whole divisions or executive directors that coordinated the entire COVID-crisis response in their jurisdiction. All participants were significantly involved in the coordination of the COVID-vaccination campaign in their jurisdiction.<br>Educational backgrounds of participants included: medicine, pharmacy, social studies, law, public administration, crisis management, logistics, business, economics, epidemiology, health sciences, philosophy, evaluation, biology, political sciences, pedagogics.
Data collection		
17. Interview guide	Were questions, prompts, guides provided by the authors? Was it pilot tested?	The interview guide was developed based on a review of the literature (policy guidance from the German Federal Ministry of Health and the WHO as well as peer-reviewed journal articles on the COVID-19 vaccination roll-out).<br>The guide was reviewed by study authors SD, OW, FS, DA and 4 external experts.<br>The interview guide was pilot tested with 1 person familiar with the subject and 2 people unfamiliar with the subject.
18. Repeat interviews	Were repeat interviews carried out? If yes, how many?	No.
19. Audio/visual recording	Did the research use audio or visual recording to collect the data?	Interviews were audio recorded.
20. Field notes	Were field notes made during and/or after the interview or focus group?	Field notes were taken and served as input for developing themes and codes.
21. Duration	What was the duration of the interviews or focus group?	The interviews lasted from 30 to 97 minutes with an average duration of 58 minutes.
22. Data saturation	Was data saturation discussed?	Data saturation was discussed specifically with regards to collecting input from all 16 federal states as some similarity in terms of experiences and inputs was to be expected from similar "types" of federal states, e.g., by size, structure (rural, urban) and historic region (new versus old federal states).
23. Transcripts returned	Were transcripts returned to participants for comment and/or correction?	Select quotes and data points, e.g. , the number of vaccination centers used, definition of vaccination center versus vaccination point (“Impfstelle” in German), were returned to participants for review and clarification.
**Domain 3: analysis and findings**		
Data analysis		

24. Number of data coders	How many data coders coded the data?	2 coders. SD and DA each coded a sample of 20% of interviews. The interrater-reliability score was 0.85 demonstrating a high overlap between raters and reliability of coding. SD then coded the remaining interviews.
25. Description of the coding tree	Did authors provide a description of the coding tree?	Yes. Overarching categories and recurring themes were used as the basis for creating coding templates.
26. Derivation of themes	Were themes identified in advance or derived from the data?	Combination of inductive and theory-driven coding: Some categories and themes were identified in advance through literature review (theory-driven); others were derived from the data.
27. Software	What software, if applicable, was used to manage the data?	Transcription Software (Trint)<br>Microsoft Office tools (Word, Excel)
28. Participant checking	Did participants provide feedback on the findings?	24 of 27 experts provided feedback on select quotes and data sent to them for clarification.
Reporting		
29. Quotations presented	Were participant quotations presented to illustrate the themes/findings? Was each quotation identified? e.g. participant number	Anonymous quotations using the overarching function of the participant were used to illustrate the findings.<br>Due to the highly politicized environment, maintaining the anonymity of the interviewees was essential to enable an open and honest exchange.
30. Data and findings consistent	Was there consistency between the data presented and the findings?	Yes.
31. Clarity of major themes	Were major themes clearly presented in the findings?	Yes.
32. Clarity of minor themes	Is there a description of diverse cases or discussion of minor themes?	Yes.

Reflexivity statements

<u>Reflexivity statement SD</u>

SD conceptualized the study, established the methodology, conducted and coded all interviews. Prior to her doctoral research, SD could be classified as insider. At the time of conducting the interviews and analysis, she held the position of a well-informed outsider.

SD holds an undergraduate degree in Political Science, History and Economics (BA) and a graduate degree in Health Policy and Health Economics (MSc). She has been professionally active in the field of health policy and healthcare innovation for more than seven years. Before taking on her position as doctoral researcher studying vaccination centers and national vaccination campaign implementation, she was involved in national COVID vaccination campaign coordination as an employee of the federal ministry of health. She furthermore supported special operations for two COVID vaccination centers in Berlin and organized a COVID vaccination day in a Bavarian town.

SD hence had privileged access to the field as she was familiar with the technical terminology, the proceedings, and the involved stakeholders. It allowed her to more easily reach some of the experts as she knew them through a prior professional working relationship (N=10 of 27 participants). It also helped with finding contact persons or liaisons in all institutions. Nonetheless, where the experts or liaisons were not directly known, especially at the federal state level, reaching the interview partners sometimes was a lengthy process. Due to the political nature of the topic and the time-consuming nature of the COVID-response work, some experts were not immediately available and only agreed to participate upon multiple requests or with further information on the objectives of the research project.

During the interviews, SD could often establish a trusted researcher-researched relationship as she could demonstrate familiarity with the field of research where experts were not personally known. Personal rapport may also have led some interview partners to speak more openly. It can also be noted that for few very senior interview partners that were not personally known to the researcher, the tone was very official. Interview partners were overall cooperative and demonstrated flexibility in spending additional time where necessary to provide information on all questionnaire items. Over the course of the interviews, the order of the questions could be changed. Using an interview guide however, ensured, that all key questions were asked across interview partners. Through a somewhat shared experience with interviewees, SD was able to ask pertinent follow-up questions, particularly on themes that were less debated in the community. Some of the processes and particularities, especially at federal state level, were not known to SD, which led her to ask clarifying topics where topics were particularly local.

Her prior experience helped SD to quickly develop categories and themes informed by literature review and her own experience in the field. This familiarity benefited the data collection and analysis as it served as a "reality-check" for novelty. This more insider perspective was balanced by the co-coder's outsider perspective. The co-coder could call attention to themes that may have seemed obvious to SD.

<u>Reflexivity statement DA</u>

DA helped establish the methodology and coded five interviews. DA did not participate in the interview process. DA only received pseudonymized interview transcripts, which was the sole basis of his analysis of the data. None of the interview partners were known to DA. At the time of research, DA held the position of informed outsider.

DA has a background in management sciences (BSc), international health policy (MSc) and health services research / health economics (PhD). DA has professional working experience in the private and public sectors of the German healthcare system, focusing on the field of healthcare innovation through data-driven solutions. He is hence familiar with regulatory frameworks, incentive structures as well as the political and regulatory milieu of the German healthcare system.

DA has experience conducting interview research using thematic analysis in the field of health policy in particular (e.g., see https://pubmed.ncbi.nlm.nih.gov/29301149/). This helped him in contributing to the development of the methodology and helped with developing the coding process.

During the coding process, his background in health services research and healthcare provision enabled him to ask probing questions to SD to ensure the content was understandable to informed, but not intimately familiar readers. Importantly, DA added an outside perspective to the coding process, highlighted themes and quotes that may not have stood out to SD due to her continuous exposure to the topic. Furthermore, DA's understanding of health policy provided a foundational lens through

which he could contextualize the research findings. Although he did not have firsthand experience with the vaccination campaign, his grasp of the broader policy implications and intricacies of the German healthcare system permitted him to contextualize, question, and validate the themes that emerged.

<u>Reflexivity Summary</u>

The researchers are aware that in qualitative research, thematic analysis and interviewing there is an inherent risk of researchers projecting their beliefs, perceptions and biases during the research process. While SD's intimate knowledge of the research field may be helpful in establishing rapport and quickly distinguishing relevant themes it may also carry elements of operational blindness, which is why it was counterbalanced by DA's outsider researcher perspective. Overall, in order to reduce bias and the imposition of personal beliefs and perceptions several precautions were taken: semi-structured interview guides were used in all interviews, tested and discussed with all researchers and external experts; two separate coders; vertical and horizontal analysis, in particular comparing inputs from across different federal states; where necessary verification of select details with participants and through gray literature review.

Researcher triangulation formed the basis of the entire research. The research team consisted of members deeply familiar with the topic (SD and OW) and members not deeply familiar with the topic (DA and FS). The research team contributed broad methodological and disciplinary perspectives and experiences and could challenge each other's assumptions. The questionnaire, coding, the results, the interpretation and use of quotes were discussed iteratively among the researchers. Critical review and discussion among the researchers also reduced the likelihood of withholding information which may be assumed obvious to those intimately familiar with the topic, e.g., technical terminology or specific processes.
