# Supplementary material for: The Role of Vaccination Centers in a National Mass Immunization Campaign—Policymaker Insights from the German COVID-19 Pandemic Vaccine Roll-Out
Source: Vaccines (Basel). 2023 Sep 30;11(10):1552. doi: 10.3390/vaccines11101552 (PMC10611148; doi:10.3390/vaccines11101552)
Supplement: Supplementary file 1 [file vaccines-11-01552-s001.zip › S2_Supplement_S2_Semi-structured interview guide_vF_proof.pdf]

S2. Semi-structured interview guide for crisis / vaccination roll-out coordinators at federal and state level ministries of health and stakeholder representatives at federal level in Germany

May 2022

The present publication focuses on topics i, ii, iii, and v.

**Topics**	**Example Questions and Reminders**
**Background and Introduction**	• Brief introduction of the research project and the interviewer<br>• Confirmation of voluntary participation and anonymity of the evaluation<br>• Overview of the interview plan<br>• Overview over the role and operational responsibilities of the interviewee during the COVID-19 vaccination campaign
**i. Objectives and success criteria for a national mass vaccination campaign**	• What are the objectives of a national pandemic vaccination roll-out? When is a national pandemic vaccination roll-out successful?<br>• How do you rate the decision to use vaccination centers in the COVID-19 vaccination campaign?<br>• How did vaccination centers contribute to the success of the vaccination campaign in Germany?
**ii. The use of vaccination centers compared with other vaccination structures**	• When and where is the use of vaccination centers particularly advantageous?<br>• In contrast, when is the use of other vaccination structures (mobile vaccination teams, medical practices, company physicians, pharmacists, dentists, veterinarians) particularly useful?<br>• How do you evaluate vaccination centers in comparison to other vaccination structures, especially doctors' offices and company physicians or company medical services? What advantages and disadvantages do vaccination centers offer in comparison?<br><br>• When is the right time to move from centralized to decentralized vaccine delivery?<br>• Was the transition time chosen the right one?<br><br>• How and where should you vaccinate against COVID-19 in the future?<br>• What would have to happen for you to consider setting up vaccination centers again? Are there any specific indicators?
**iii. Pandemic Preparedness and long-term use of vaccination centers**	• What is the long-term role of vaccination centers? (E.g., in flu epidemics, school immunizations, pandemic, ..)<br>• What can other sectors learn from the "vaccination center concept"?<br><br>• What arrangements can be made to quickly make vaccination centers operational again?<br>• Is your organization making such arrangements?<br><br>• Is your organization conducting a lessons learnt process of the vaccination campaign?
**iv. Coordination and organization of the vaccination campaign**	• Who was overall responsible for managing the COVID-19 vaccination campaign in your state / at federal level?<br>• Which other stakeholders were involved in the organization and implementation of the vaccination campaign?<br>• How were stakeholders involved (through which fora, mechanisms, …)?

	• Who was overall responsible for managing the COVID-19 vaccination centers in your state / at federal level?<br>• Which other stakeholders were involved in the organization and implementation of the vaccination centers?<br>• How were stakeholders involved (through which fora, mechanisms, …)?<br><br>• How would you rate the coordination of the vaccination campaign?<br>• How would you specifically rate the coordination between vaccination centers and other vaccination structures (medical practices, company physicians)?
**v. Best practices and areas for improvement for management, coordination and implementation of the vaccination campaign**	• What went particularly well with regards to the overall immunization campaign? (would be a best practice)?<br>• What would you do differently in relation to the overall immunization campaign (would be an area for improvement)?<br><br>• What went particularly well in relation to the vaccination centers?<br>• What would you do differently in relation to the vaccination centers?
**Closing**	• Time for additional remarks<br>• Thanks
