# Supplementary material for: The Role of Vaccination Centers in a National Mass Immunization Campaign—Policymaker Insights from the German COVID-19 Pandemic Vaccine Roll-Out
Source: Vaccines (Basel). 2023 Sep 30;11(10):1552. doi: 10.3390/vaccines11101552 (PMC10611148; doi:10.3390/vaccines11101552)
Supplement: Supplementary file 1 [file vaccines-11-01552-s001.zip › S3_Supplement_S3_Table_Additional Quotes from Experts_vF_proof.pdf]

S3. Additional quotes from federal and state level coordinators and federal level stakeholder representatives on the role of vaccination centers in the German COVID-19 vaccination roll-out

Section	Context	Quotes
3.1. Target criteria for a successful vaccination campaign	Vaccination Coverage	*"The vaccination roll-out is successful, when every person that wants to get vaccinated has gotten vaccinated."* (state level coordinator)<br>*"The vaccination rate was what the political level and public always looked at, which I thought was a bit of a shame, because that doesn't reflect everything.*" (state level coordinator)<br>*"In the end, the success of the vaccination campaign can only be measured by the vaccination rate. Whereby one must bear in mind that there has been a quite stable group of opponents of vaccination in Germany for more than 100 years. This distinguishes us from other European countries*." (state level coordinator)
	Trust and acceptance from the population	*"In the beginning getting buy-in for the prioritization, for who gets to get vaccinated at what time and how this group will grow based on availability of vaccines, was an important goal.*" (federal level coordinator)<br>*"Registered and accepted by the population."* (federal level coordinator)<br>*"This includes the acceptance that part of the population may not want to get vaccinated."* (federal level coordinator)<br>*"Of course, we want people to get vaccinated. But having an educated population that deliberately decides not to get vaccinated could also mean that the campaign was successful. The success consists in the population being knowledgeable and informed about the vaccines."* (federal level coordinator)
3.2.1. Advantages and disadvantages of public vaccination centers in comparison to regular vaccination structures	Controllability	"*Via the vaccination centers, we could ensure that there was enough vaccine for the second dose of the vaccination course.*" (state level coordinator).<br>"*When the vaccination campaign started in the outpatient practices, it was amazing how many people were suddenly vaccinated, even though it wasn't their turn yet according to the prioritization.* [...] *The legitimate and necessary prioritization would have failed in a purely outpatient setting*" (federal level stakeholder representative).<br>*„The prioritization would have failed in the medical practices* [...] T*he advantage of the vaccination centers was that they were neutral in the selection of patients. If a long-term patient asks whether they can get vaccinated, it would be difficult for their physician to say no."* (federal level stakeholder representative)
	Organization	*"The quality of the service is much higher [in the vaccination centers] than in any other structure in terms of data collection, emergency management, the handling of vaccination documentation by the pharmacists. A medical practice cannot provide comprehensive quality management in this form."* (state level coordinator).<br>*"At vaccination centers, you don't find waiting rooms full of sick people." (state level coordinator)*<br>*"Outpatient medical practices are accustomed to vaccinating."* (federal level stakeholder representative)
	Logistics	*"From my point of view, there must be a central management in such a situation [...] The granularity that was subsequently added through the supply of medical practices makes such a system highly complex and highly vulnerable from a logistical point of*

		view. You suddenly have far too many stakeholders involved in the game, all of whom somehow register needs that then have to be met." (federal level coordinator)<br><br>*"From a logistical point of view or a military point of view, a simple system is always more stable than a complex system. Complex systems always have more vulnerabilities or the possibility of vulnerabilities. I actually would have liked us to wait [with decentralization] until we reach the 60-70% [vaccination rate]."* (federal level coordinator)<br><br>*"The vaccination centers make logistics easier. If you go to one or a few centers, that is of course easier than hundreds of medical practices."* (state level coordinator)
	Throughput (Speed & Volume)	"*The doctor's office can't say 'I'll be closed for 14 days' and only vaccinate. Other patients would remain untreated*". (federal level stakeholder representative).<br><br>*"The vaccination centers did nothing else [apart from vaccinating] all day. This also allows you to work efficiently."* (state level coordinator).<br><br>"*The speed of different structures is difficult to assess [... it is] distorted by distribution and vaccine availability at different points in time*". (federal level coordinator)
	Accessibility and Openness	*"It is less effort [getting a vaccination at a vaccination center] than finding a doctor who offers vaccinations because he only fills the syringes once a week or [...] only offers vaccinations to his own patients."* (state level coordinator).<br><br>*"The accessibility of the vaccination center was certainly better than that of the doctor's offices, because they were open longer and people could then go during off-peak times when no doctor's office was open. The actual distance between the place of residence and the vaccination site is of course better with the doctor's office."* (state level coordinator)<br><br>*"You need to reach people where they already are."* (state level coordinator)<br><br>*"We're not that big a federal state in terms of population, but we also have a large surface area to cover, and we wanted to make sure that no one who wanted to get vaccinated had to take an hour-long train ride to the nearest city. We tried to spread the vaccination sites well across the state to achieve low-threshold access and give many the incentive to get vaccinated."* (state level coordinator)<br><br>*"The low-threshold accessibility of the vaccination centers is really extremely positive because there is a non-negligible portion of the population that doesn't have a primary care physician at all."* (state level coordinator)
	Physical Accessibility	*"If people can drive ten kilometers on a Sunday morning just to get fresh bread rolls, then they can also drive ten kilometers to get vaccinated."* (federal level stakeholder representative)
	Personal relationship	*"A vaccination center is an anonymous entity. Doctors also work there, but not the familiar faces."* (state level coordinator)<br><br>*"The vaccination centers also enjoyed a great deal of trust among the population. I find this trust in local structures and also trust in the state, which suffered in many cases during the Corona pandemic, quite remarkable."* (state level coordinator)<br><br>*"Trust is a very individual question. There was no feedback to suggest that there was less trust in the vaccination centers, very positive."* (state level coordinator).<br><br>"*Physicians in outpatient medical practice can evaluate and apply the topic of prioritization differently using their regular care provision viewpoint, perhaps somewhat less formalized and more from their experience with their patients.*" (federal

		level coordinator)
	Cost	*"In the beginning, there were these debates in the world of statutory health insurance physicians about how much should be paid for a vaccination."* (state level coordinator)<br><br>*"Cost-effectiveness is difficult to evaluate because many factors cannot be taken into account, and it is therefore actually a secondary factor. I can calculate the costs of one vaccination, but what is not considered are follow-up costs that are not directly noticeable or cannot be quantified because, for example, employees cannot work because they wait a long time for a vaccination or are in quarantine for longer."* (state level coordinator)
	Symbolism	*"[The vaccination centers] were a symbol visible to the outside world: Something important is happening here! That's why the placement in the city center is important. People see quite obviously: There are vaccinations, people stand in line. I see that something happens, I see orderly, nice queues, the people look so similar to me. Oh look, back there is the exit and they all survived. [...] We have to tell a story. We have to make this story believable. And a vaccination center is a place where things get serious, where you can see that something is happening."* (state level coordinator)
3.2.2. Deployment of public vaccination centers for national pandemic vaccination roll-out	Deployment Criteria	*"Whenever a limited resource needs to be allocated in a targeted way, with a prioritization, it will be most effectively managed through centrally controlled vaccination facilities, i.e,. through quasi-governmental control."* (state level coordinator)<br><br>*"The participation of state structures is only justifiable if there is an urgent medical need or if strict prioritization has to be enforced."* (state level coordinator).<br><br>*"Decentralization is warranted when distributive justice does not have to be brought about through administrative measures anymore."* (federal level stakeholder representative).<br><br>*"You need a certain critical quantity of vaccines to be able to distribute it into the periphery."* (state level coordinator)<br><br>*"In the beginning, we were all still inexperienced with this type of vaccine; it was carried around like the holy grail, so that it would not be shaken, stirred or mechanically stressed in any other way. These were already complex requirements, and it would not have been possible to distribute it broadly at this point in time."* (state level coordinator)<br><br>*"The vaccination centers were very important, especially in rural areas, where the density of doctors is not that high."* (state level coordinator).<br><br>*"In addition to vaccinations, in addition to Corona, there were also normal sick people. So, it was good that we created the parallel structures. [...] doctors would have been overrun*." (coordinator state level).<br><br>*"Whatever the doctors themselves say, they were very happy that there was a government vaccination offer. Because […] the doctor's practices were heavily burdened by the whole pandemic if they offered outpatient care. During the Omicron variant in particular, it was noticeable that not only the burden from the patients was there, but that some of the doctor's office staff were also absent."* (state level coordinator)<br><br>*"That's related to funding. There is a commitment from the federal government to finance half [of the vaccination center expenditure]. The federal states can't finance something like this by themselves."* (state level coordinator)

	Transition Timing	*"I believe that this distributed, phased system that has been implemented now, depending on the availability of vaccines, is a sensible system. It also needs all three [structures - vaccination centers, resident doctors, company doctors]."* (state level coordinator)<br><br>*"The vaccination campaign speed really picked up when the federal government involved the outpatient physician practices. But they could only do that once sufficient vaccines were available."* (state level coordinator)<br><br>*"For a while, the number of telephone calls in the doctor's offices was no longer manageable."* (state level coordinator).<br><br>*"At the time, this was problematic as there was still too little vaccine on the market. [...] We had established the vaccination centers that could administer a large number of vaccinations, but then we pulled the vaccine doses and distributed them to another area. As a result, the vaccinations that had already been paid for in the vaccination centers were not used and then paid for again in the medical practices. So that doesn't make sense if I have a large structure that has high fixed costs and which I then don't use to capacity."* (state level coordinator)
	Changing Role of government immunization services	*"Everything has to interlock somehow."* (state level coordinator)<br><br>*"We generally see vaccinations in the outpatient sector and see ourselves as a supporting instrument. At the very beginning it could not be implemented any other way due to pharmaceutical requirements."* (state level coordinator).<br><br>*"I think the parallelism of both structures [vaccination centers and doctor's offices] was important and necessary. Absolutely."* (federal level stakeholder representative)<br><br>"*I consider both structures [vaccination centers and general practitioners] to be important, they are complementary structures because the needs differ in terms of the type of medical care and accessibility*." (federal level stakeholder representative)
3.3. Additional take-aways for best practice vaccination center roll-out	Flexible design of government vaccination structures adapted to circumstances	*"The mobile vaccination teams have been used to drive regional vaccination offers and vaccination campaigns again and again."* (state level coordinator)<br><br>"*In the course of the vaccination campaign, it would have been good to be more fragmented and to get closer to the population. The large vaccination centers do not offer any flexibility*." (state level coordinator)<br><br>*"In rural areas, you don't need huge vaccination centers now. So, it's really important that you either set up smaller vaccination centers or mobile units where you say, 'okay, we're going from market place to market place now and try to reach people there while they're buying their lettuce'"* (federal level coordinator)<br><br>*"Large 'bulk processing' vaccination centers are the better model when the vaccine is in high demand. I call this the 'come-here model'. If the demand situation changes [...] then you cannot trust that people will make their way to the vaccination center. It is then better to organize offers where they are anyway - ['go to model']."* (state level coordinator).<br><br>*"I think it's better to have multiple vaccination sites that are closer to the people."* (state level coordinator)<br><br>*"Because of this huge area, we established [...] units, so to speak, small vaccination centers, which were open for a week and then closed for two weeks or something similar. [...] not such large vaccination centers like in Hamburg or Berlin, but just a smaller base."* (state level coordinator)<br><br>*"The vaccination units are much smaller and more regional, making it easier for us to control and scale accordingly. We have seen over the summer of 2021 that interest in*

		vaccination had waned and vaccination centers were not as busy as they could have been. And with these smaller units, on the one hand you are closer to the people and on the other hand we can react more flexibly when the demand subsides." (state level coordinator)<br><br>*"These were really huge centers for real mass flow. But I can well imagine something three sizes smaller [...] that something like this could be set up quickly, just like the test centers, perhaps [you could] also provide the vaccinations in such a structure. [...] a mobile station, something between a vaccination center and mobile teams. [...] smaller, more mobile, more punchy."* (federal level coordinator)
	Scalability and optimized use of available vaccination capacities	"*The vaccination centers shouldn't have been closed temporarily. The structures could have been scaled up and down like we are doing now: keep the physical infrastructure and scale the staffing numbers up and down. [Keeping the structures in standby, scalable] is costly of course, but it was even more costly in the end to lay everyone off, dismantle everything, to then find new spaces, rehire, and rebuild. That's ultimately more expensive than establishing a structure once that sort of breathes*." (state level coordinator)<br><br>"*In the beginning, the vaccination centers were partly empty because the vaccine was not there. Then there was enough vaccine. But then people went to their family doctor rather than to the vaccination centers.*" (federal level coordinator)<br><br>"*The funds that are being put into the vaccination centers could also be put into mobile vaccination teams so that they can visit refugee homes, for example, and offer vaccinations with low barriers.*" (federal level coordinator)<br><br>*"The offer must actually be based on the actual demand*." (state level coordinator)
3.4 Post-COVID: Transfer and operational readiness of the concept mass vaccination center	Long-term use	*"Generally, for crises that occur at short notice, are of a larger nature and cannot be managed with the normal civil protection units [...] alone. Above all, this short-term approach is important in order to solve large tasks at short notice."* (state level coordinator).
	Operational readiness	*"Everyone seemed to run their proper "youth research project". In parts, it was really impressive [...] I think it would be good if there was a blueprint for it - A blueprint 'How do you build a model vaccination center?'"* (federal level coordinator)<br><br>"*There needs to be a list of basic structures, a kind of checklist, which can then be used as a guide. For example, this was done by the Federal Ministry of Health for the conception of the vaccination centers. We then modified it a bit for our purposes.*" (state level coordinator)<br><br>*"The property search cost us an awful lot of time in the beginning. We had enough time because there was no vaccine, but for the future I think it's good if you identify certain sites in the community upfront and they are already known.*" (state level coordinator)<br><br>"*It's always easier to build upon something than to establish something from scratch [...] I think all municipalities and federal states would be well advised if they had a basic structure in place.*" (state level coordinator)<br><br>*"We would handle [provisioning] flexibly with the municipalities, because we don't know whether the next crisis will also affect the entire state or just a specific one."* (state level coordinator)<br><br>*"You can always find sites. That almost always results from the circumstances of the respective federal state [...] in the worst case there is another summit [of political decision-makers] and then something is quickly built up from scratch."* (state level coordinator)

		“We are in the process of setting up a state material warehouse, where we will have a supply for about three months in case there are shortages on the market again." (state level coordinator)<br><br>*“The vaccination center is set up quickly. The problem is the people needed to operate them [...]. You have to find the right people [...] And they also want to have some certainty as far as their nearer future is concerned.”* (state level coordinator)<br><br>*“We actually lack crisis managers who want to design and build such facilities properly. [...] this is not a temporary flood response, but the creation of a health structure within a very short time [...]. This has never been considered in crisis management, above all not that it would last for months.”* (state level coordinator)<br><br>*“It would be nice if there were vaccination centers everywhere in the public health service. Other types of vaccinations that are carried out by the public health service. That should be resuscitated.*" (state level coordinator)<br><br>"*Overall, we have to make sure that we are crisis-proof.*" (state level coordinator).<br><br>"*[We should not] see medical disaster preparedness as a side issue.*" (state level coordinator)
